# Anterior Intercondylar Notch Geometry in Relation to the Native Anterior Cruciate Ligament Size

**DOI:** 10.3390/jcm13020309

**Published:** 2024-01-05

**Authors:** Eduard M. Cernat, Alina Dima, Claudiu Popescu, Andrei Neagu, Cezar Betianu, Marius Moga, Loredana Sabina Cornelia Manolescu, Adrian Barbilian

**Affiliations:** 1 Department of Clinical Education, Faculty of Medicine, Carol Davila University of Medicine and Pharmacy, 020021 Bucharest, Romania; eduard-marcel.cernat@drd.umfcd.ro (E.M.C.); marius.moga@umfcd.ro (M.M.); adrian.barbilian@umfcd.ro (A.B.); 2 Department of Orthopedics, Dr. Carol Davila Central Military University Emergency Hospital, 010242 Bucharest, Romania; 3 Department of Reumatology, Colentina Clinical Hospital, 020125 Bucharest, Romania; alina_dima@outlook.com; 4 Department of Reumatology, Dr. Ion Stoia Rheumatic Disease Center, 030167 Bucharest, Romania; 5 Department of Radiology, Dr. Carol Davila Central Military University Emergency Hospital, 010242 Bucharest, Romania; andrei-valentin.neagu@rez.umfcd.ro (A.N.); cezar.betianu@icloud.com (C.B.); 6 Department of Fundamental Science, Carol Davila University of Medicine and Pharmacy, 020021 Bucharest, Romania

**Keywords:** anterior cruciate ligament, intercondylar notch, knee injuries, lateral trochlear inclination, notch shape

## Abstract

Background: The intercondylar notch (ICN) and the anterior cruciate ligament (ACL) are important structures in knee morphometry, with key roles in stabilizing the knee. Aim: To determine the associations between the specific shape of the ICN (A-, W-, or U-shape) and the ACL size in patients with intact ACLs. Methods: Magnetic resonance imaging (MRI) scans were independently analyzed by two experts: one orthopedic surgeon and one imaging physician. In all cases, the following measurements were taken based on the existing definitions: ACL area, anterior ICN (aICN) area, ICN width, lateral trochlear inclination (LTI), and Insall–Salvati index. Results: A total of 65 cases (50.8% male; 33.8 ± 10.2 years mean age at inclusion) were included in the study. The ACL and aICN areas were significantly larger in patients with U-shaped compared with A-shaped and W-shaped ICNs: 0.50 (0.20–0.80) vs. 0.40 (0.20–0.80) vs. 0.40 (0.30–0.80), *p* = 0.011 and 1.16 (0.57–3.60) vs. 0.47 (0.15–0.95) vs. 0.37 (0.15–0.81), *p* < 0.001, respectively. Internal meniscal lesions were more common in cases with U-shaped ICNs (64.0%), while external ones were more common in W-shaped ICN cases (35.3%). None of the A-shaped cases had external chondral or meniscal lesions. The ACL area was significantly larger in males and internal meniscal injuries, with no differences between chondral lesions, external meniscal injuries, patellar chondral lesions, patella alta, or trochlear dysplasia. Conclusion: The specific shape of the intercondylar notch was associated with the anterior cruciate ligament–anterior intercondylar notch (ACL–aICN) area size correlation, with a strong correlation between ACL and aICN area when the intercondylar notch was A-shaped or W-shaped, and a low correlation when the notch was U- shaped. The specific shape of the intercondylar notch (A-, W-, or U-shape) was associated with the occurrence of both internal and external meniscal injuries, with the U-shaped intercondylar notch morphometry being more frequent in cases with internal meniscal injuries and the W-shape being more common in cases with external meniscal injuries.

## 1. Introduction

The morphometric anatomy of the knee, mainly characterized by the characteristics of the anterior cruciate ligament (ACL) and the intercondylar notch (ICN), has been proposed as a risk factor for non-contact knee injuries [1]. Meniscus injuries are one of the most common orthopedic issues worldwide [2]. The knee meniscus and the anterior cruciate ligament (ACL) are closely associated with the knee physiologic morphometry [2].

Meniscal injuries are common injuries at almost any age; the annual incidence is 61 per 100,000 individuals [3] and they may precede or succeed anterior cruciate ligament injuries. Meniscal injury induces degenerative changes in the knee joint. The meniscus assures the stability of the knee, distributes axial load, and is crucial for proprioception and lubrication of the joint [4]. Due to its many important roles in the knee joint [5], preventing and predicting meniscal lesions is important [6].

Another factor that causes knee instability is ACL lesions. The anterior cruciate ligament is the main restraint of anterior tibial translation [7]. Many factors may contribute to its tearing, including intrinsic and extrinsic factors [8,9]. While extrinsic factors may vary greatly and sometimes are difficult to control, intrinsic factors can be assessed and can play a role in the prediction of predisposition to injury. The most studied intrinsic factors are variations in ACL size, variations in the shape of the tibial plateau, and morphometry of the femoral intercondylar notch [10].

Carrying unexpectedly heavy loads, exercising with high intensity, or involvement in traffic accidents may cause evident injuries to ACLs or menisci [11]; however, the development of knee pain in an individual with no obvious external involvement raises questions about the individual’s morphometric anatomy of the knee. Therefore, knowing the predisposition for meniscus or ACL lesions in an individual with non-contact knee pain would be extremely useful for determining future options for treatment or monitoring.

There is a paucity of data exploring the morphometric anatomy of the knee in individuals with non-contact knee pain compared with post-traumatic cohorts of patients [12]. Nevertheless, the timepoint of a return to normal life and sports after anterior cruciate ligament (ACL) reconstruction is complex and depends on many factors [13,14].

The aim of this study was to assess the associations between the intercondylar notch cross-sectional area and the anterior cruciate ligament cross-sectional area in different notch shapes, as well as the association of notch shape with structural changes in patients who underwent knee magnetic resonance imaging (MRI) for non-contact knee pain. To the best of our knowledge, this is the first study that aims to correlate the anterior part of the ICN with the size of the ACL. Previous studies focused on the global aspect of the ICN or the ICN width and its correlation with the risk of injury or ACL anatomy.

## 2. Materials and Methods

### 2.1. Study Population

This is a cross-sectional single-center retrospective study of patients who underwent knee MRIs in our department between September 2019 and September 2022. We reviewed all medical files and included all adult patients (more than 18 years old) with non-contact sources of knee pain.

The following exclusion criteria were applied: history of trauma; history of prior surgery or history of fracture in the knee region; over 50 years of age at the time of evaluation; and presence of morphologic abnormalities like arthritis, meniscal extrusion, and anterior cruciate ligament injury. Cases where the depicted part of the length of the femur and tibia were less than 5 cm were also excluded (as 5 cm is the minimum length needed to draw the longitudinal axes of these bones). No professional athletes were included in our study, albeit not on purpose. We did not include the weight during data collection, although the weight limit for the MRI in the department is 100 kg for tall patients (>1.85 m) and decreases for patients that are less than 1.80 m tall. We consider this height–weight correlation (body mass index, BMI < 30) as representative of the general population.

### 2.2. MRI Acquisition

**Figure 1:**
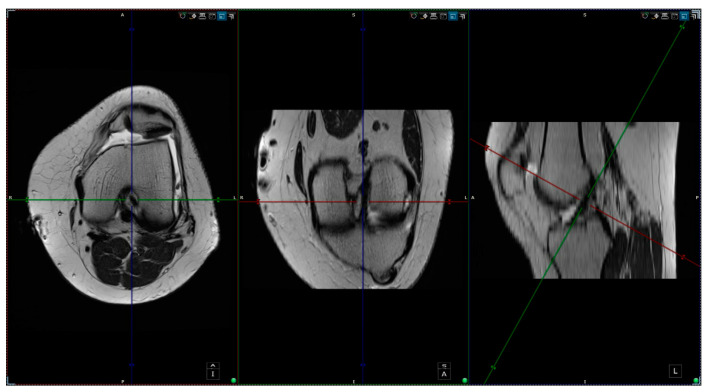
Custom Axial Plane (CAP).

Two 1.5-T magnets (Magnetom Essenza and Magnetom Aero, Siemens Medical Solutions; Siemens Healthineers Headquarters, Forchheim, Germany) were used to perform the MRI examinations following similar protocols. Both obtained 3-mm slices of the knee using three standard orthogonal techniques (axial, coronal, and sagittal) with a combination of fluid-sensitive sequences—T2-weighted (T2W), non-fat-saturated (NFS), or proton density-weighted (PDW) sequences—and T1-weighted (T1W) NFS imaging. PD-weighted sequences with and without fat saturation are usually the mainstay. It sometimes includes the T2 NFS sequence as a replacement for the PD fat-saturated (FS) in the axial plane. For our study, we mainly used T2W NFS or PDW FS true axial images, using post-processing tools to create a custom plane by applying references from the sagittal and coronal planes. The custom plane is oriented along the axis of the ACL (slightly angulated from the intercondylar roof of Blumensaat’s line) in the sagittal plane and along a line parallel to the posterior femoral condylar line in the coronal plane. As a result, the plane was perpendicular to the ACL axis and parallel to the posterior condylar line; we will call this plane the custom axial plane (CAP; Figure 1).

### 2.3. Variables and Measurements

**Figure 2:**
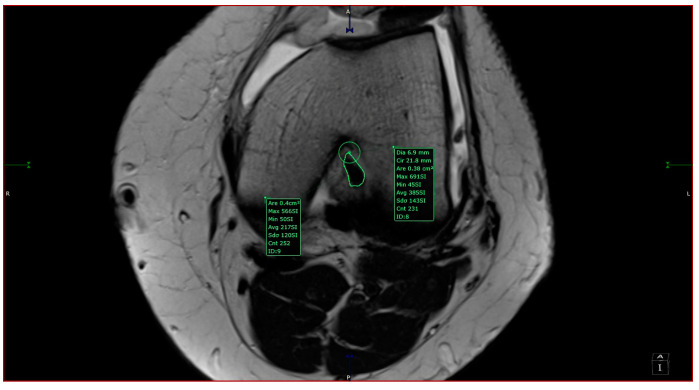
Measurements of aICN area and ACL area in the same section in an A-type notch.

The intercondylar notch was assigned to one of three categories: A-shaped, U-shaped, and W-shaped. The next step was measuring the maximum width of the intercondylar notch and its surface area in the antero-superior aspect. The area measurement was taken by drawing a circle inscribed by a cortical bone from 3 of 4 sides (anterosuperior, left, and right sides). The ACL in-plane area was measured by outlining its contour with the closed polygon tool in the soft viewer. When possible, the ACL area was measured in the same slice as the circle; if not, it was measured on a different slice with a maximum 3-slice difference (cranial or caudal). The custom plane had a slice thickness of 1 mm after post-processing, hence the variability in measurements accounts for 3 mm (±3 mm from the slice used for the measurement of aICN; Figure 2). The other sequences at our disposal were used to observe and quantitatively assess the associated findings of knee pathology. We counted as associated findings the following items: internal, external, or patellar chondral lesions; internal or external meniscus lesions; patella alta; and trochlear dysplasia. The following measurements were taken in all cases: anterior intercondylar notch (aICN) area, intercondylar notch (ICN) width, anterior cruciate ligament (ACL) area, Insall–Salvati index, and lateral trochlear inclination (LTI).

We consider that the associated MRI findings can be split into two categories:

1. Degenerative lesions (like DML—degenerative meniscus lesions—and chondral lesions) or 2. Morphological variants of the knee (like patella alta and trochlear dysplasia).

### 2.4. Measurements

**Figure 3:**
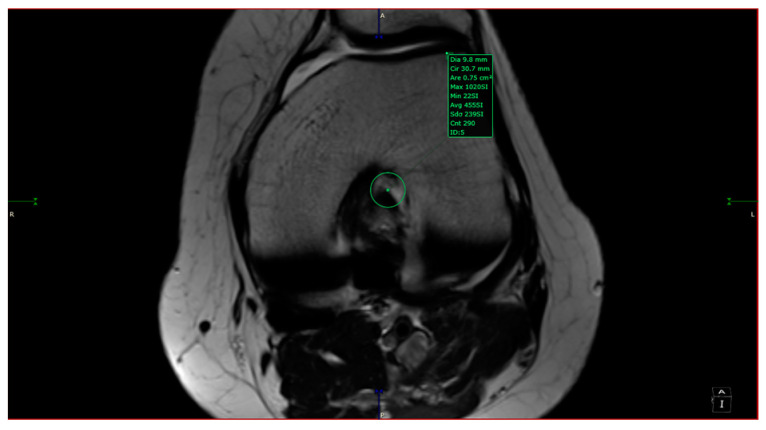
aICN area of a U-type notch.

**Figure 4:**
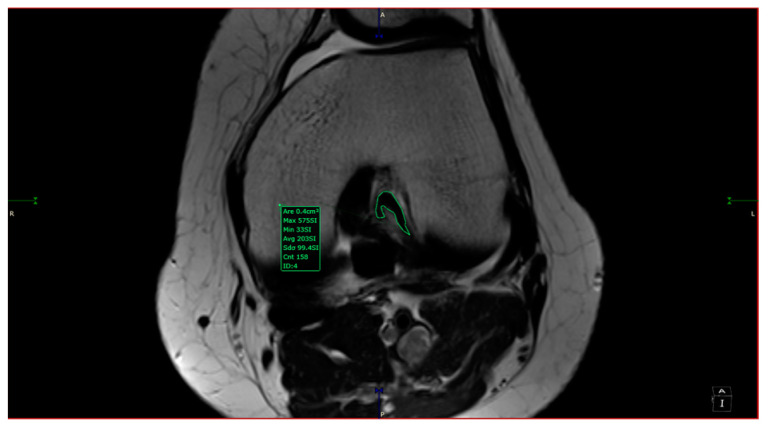
ACL area.

**Figure 5:**
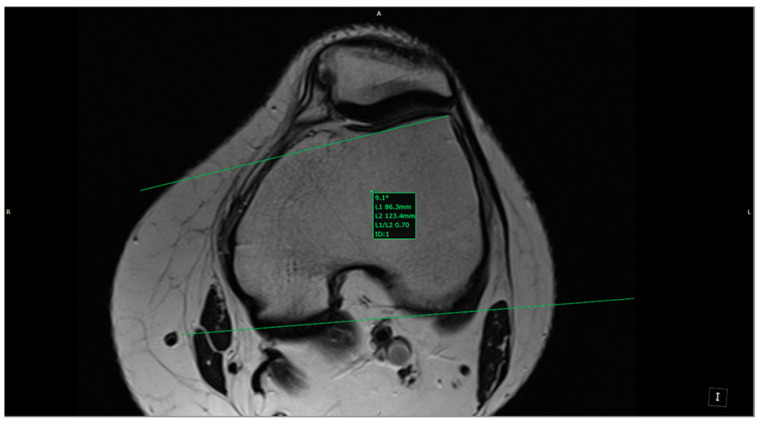
Lateral trochlear inclination.

**Figure 6:**
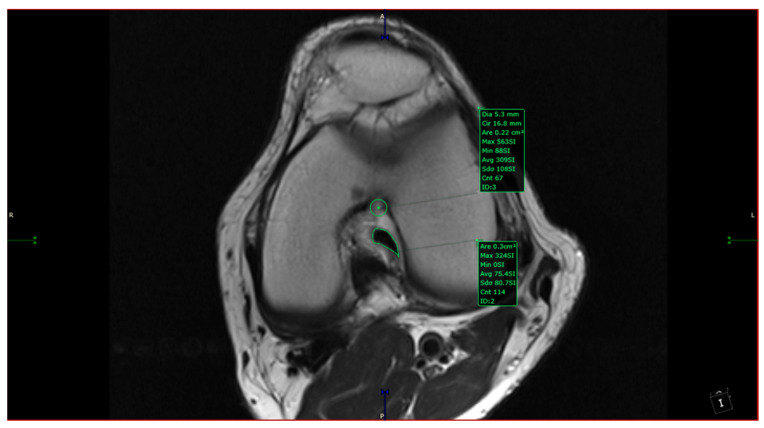
aICN area and ACL area of a W-type notch.

All the MRI scans included were analyzed by one orthopedic surgeon and one radiologist physician, each with more than 5 years of experience in knee MRI evaluation. The measurements were taken based on the following definitions:

- aICN area: area of the circle defined by three cortical walls of the antero-superior notch (in the custom axial plane/CAP; Figure 3).
- ICN width: notch width in the distal part
- ACL area: area of the closed polygon defined by pointed dots around the cross-section of the anterior cruciate ligament (in the CAP; Figure 4).
- LTI: inclination angle between the lateral femoral trochlea on the most proximal axial slice containing trochlear cartilage and a posterior condylar tangential line (in the axial plane; Figure 5).
- Patella alta: Insall–Salvati index > 1.5 (in the sagittal plane).
- Trochlear dysplasia: LTI < 11° (in the axial plane).
- aICN area and ACL area (both appear in Figure 6).

The measurements were performed by relying on a custom anatomic plane and imaging calibration of the MRI. We did not validate our method and we found it difficult to validate due to user dependence of defining the ACL area and the circle in the anterior notch. What we did was intra-operatively measure an ACL with a round section in one of the patients admitted in the study. On the MRI, we found the ACL area to be 0.38 cm^2^, while in vivo, we measured the diameter as 7 mm; this means a radius of 3.5 mm and an area of 0.3848 cm^2^ (see figure in the discussion section).

### 2.5. Statistical Analysis

Nominal qualitative variables were presented as percentages (number (%)). Continuous variables were presented as means and standard deviations for normal distributions, and medians and intervals (min–max) for non-normal distributions. Bivariate analysis using Spearman’s rho coefficient was performed for the continuous variable to identify associations like that between the intercondylar notch and ACL aria. The Mann–Whitney test was used to compare two subgroups of a dichotomous variable such as gender (male, female). Kruskal–Wallis test with post-hoc ANOVA analysis was performed to analyze more than two subgroups (e.g., the notch shape: A-, W- or U-shape). In all tests, *p*-values less than 0.05 were considered statistically significant. IBM SPSS Statistics software, version 25.0 for Windows (IBM Corp., Armonk, NY, USA), was used.

### 2.6. Ethics

Patient consent was obtained at admission and evaluation. The Hospital’s Ethical Committee approved the research (no 404/17 September 2020). All procedures undertaken in the study respected the ethical standards of the Helsinki Declaration.

## 3. Results

### 3.1. Descriptive Data: General Characteristics of the Studied Group

Magnetic resonance imaging acquisitions of a total of 65 cases meeting the inclusion and exclusion criteria were analyzed. The distribution based on gender was balanced in the study group, with 33 males (50.8%) and 32 females (49.2%). The mean age at inclusion was 33.8 ± 10.2 years. The ACL area was 0.48 cm^2^ (0.2–0.8), with higher values in males compared with females, 0.55 cm2 vs. 0.41 cm^2^, *p* = 0.002 (Table 1).

Additionally, aICN area and ICN width values were significantly higher in men compared with women, 0.96 cm^2^ vs. 0.52 cm^2^, *p* = 0.0008 and 23.87 mm vs. 20.53 mm, *p* < 0.0001, respectively, (Table 1).

### 3.2. Association between Intercondylar Notch Shape and ACL Size and Other Knee Lesions

Bivariate analysis showed that ACL was correlated with ICN in the whole group (rho = 0.66, *p*-value < 0.0001), as well as in the subgroups defined based on ICN shape (A-, W-, and U-shape). The correlation between ACL and aICN area was very strong for the A-shape (rho > 0.800), strong for the W-shape (rho 0.600–0.800), and only moderate for the U-shape (rho 0.400–0.600) (Table 2).

As presented in Table 3, the ACL area, aICN area, and ICN width values were significantly higher in cases with U-shaped ICN, while there was no relation for LTI.

The U-shape was the predominant ICN shape in males (Table 4). When comparing the occurrence of knee lesions with respect to ICN shape (A-, W-, and U-shape), the internal meniscal lesions were more common in U-shaped cases (26.1% vs. 29.4% vs. 64.0%, *p* = 0.014), while external meniscal lesions were more common in W-shaped knees (0.0% vs. 35.3% vs. 16.0%, *p* = 0.009). No significant differences were found for the internal and external chondral lesions, patellar chondral lesions, patella alta, or trochlear dysplasia occurrence (see Table 4).

### 3.3. Association between Anterior Cruciate Ligament Area and Knee Lesions

The ACL area was significantly larger in cases with internal meniscal injuries described on MRIs: 0.38 (0.23–0.69) cm^2^ vs. 0.46 (0.29–0.61) cm2, *p* = 0.002. Also, as expected, the ACL area was larger in males than in females: 0.38 (0.23–0.61) cm^2^ vs. 0.44 (0.24–0.69) cm^2^, *p* = 0.022 (see Table 5).

On the contrary, the ACL area was similar when the presence of internal or external chondral lesions, external meniscal injuries, patellar chondral lesions, patella alta, and trochlear dysplasia were studied (Table 5).

## 4. Discussion

To the best of our knowledge, this is the first study of the association between ACL and anterior notch. ACL is the most studied topic in orthopedics and one of the most studied topics in medicine in general. The association between ACL and the global notch, the lateral femoral condyle and the tibial plateau, and the meniscus has been widely studied. In this study, we assessed only the anterior part of the notch, the one that is in close contact with the ACL and focused on the association between the two structures.

From a clinical point of view, the implications can be significant. First, further studies can follow these patients and assess the risk of further lesions. This can give the chance to prevent some of the lesions and anticipate degenerative changes. As the focus on Orthobiologics has increased in recent years, further clinical trials can assess the role of orthobiologics in different knee morphologies.

The unanticipated correlation between the thickness of the ACL and internal meniscus lesions can lead to biomechanical studies that can prove the different levels of constraint of the knees with thick ACL. This is important as we aim to restore the anatomy as closely as possible when we treat an ACL lesion. The prevention protocols can be developed according to the specificity of the knee. A more constrained knee might have better proprioception compared with a less constrained knee.

Integrating the lower limb alignment in the same equation with the notch–ACL association might further challenge the deep understanding of knee anatomy and biomechanics.

A comprehensive classification of coronal plane alignment of the knee that can be used to describe healthy and arthritic knees was conducted in 2021 using preoperative soft tissue balance prediction. This classification compared kinematic alignment with mechanical alignment [14]. The results of the study showed that the distribution between healthy and arthritic patients was similar. The most common knee types described were Type II (neutral arithmetic kinematic alignment, apex distal joint line obliquity), Type I (varus arithmetic kinematic alignment, apex distal joint line obliquity), and Type V (neutral arithmetic kinematic alignment, neutral joint line obliquity). The remaining knee types were rare [14]. The study demonstrated that using the notions of varus, valgus, or neutral was incomplete as it revealed the patient’s alignment at a static moment in time and did not consider the knee joint as a whole [15,16].

Other studies with similar outcomes focused on imaging techniques to describe the morphometry of the femoral notch and its disposition on the risk of an ACL lesion [17,18,19,20,21,22,23,24,25,26,27,28]. We investigated the relationship between intercondylar notch anatomy and ACL size, patelo-femoral anatomy, meniscus, and chondral lesions. We introduce the concept of Anterior Intercondylar Notch Area (aICN area) as a different anatomical parameter, as well as ICN width or global notch size, which can be investigated in relation to the native ACL size.

### 4.1. U-Shaped Notch

**Figure 7:**
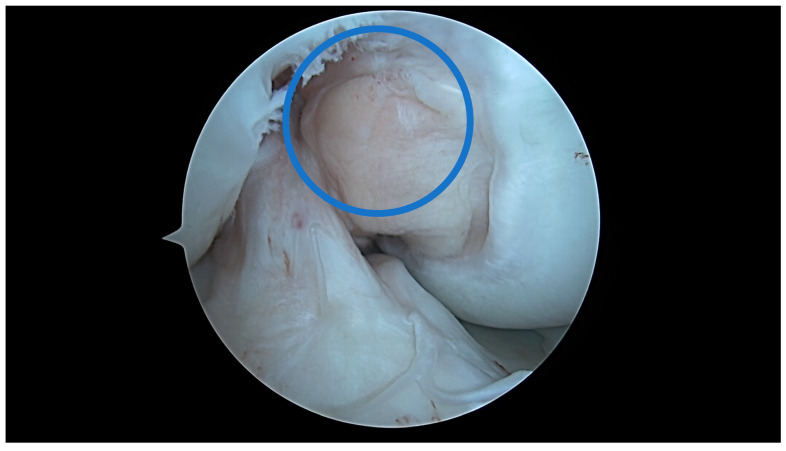
ACL–ICN relationship at 90 degrees flexion of a U-shaped notch type; blue circle = aICN area measured on the MRI.

**Figure 8:**
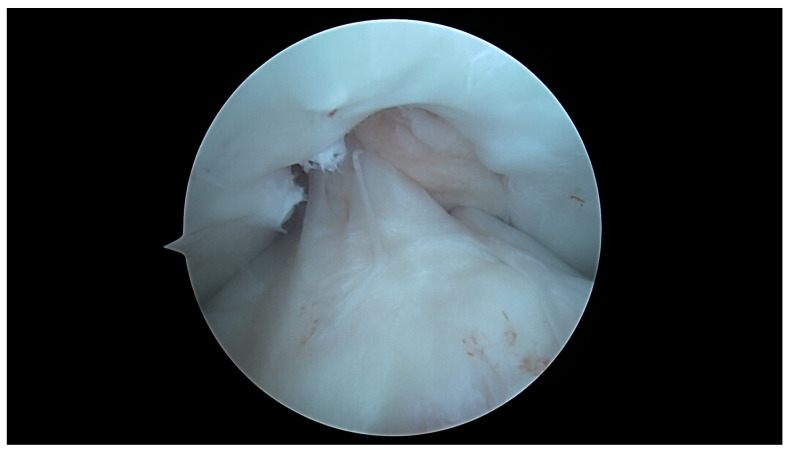
ACL–ICN relationship at 10 degrees flexion of a U-shaped notch type.

**Figure 9:**
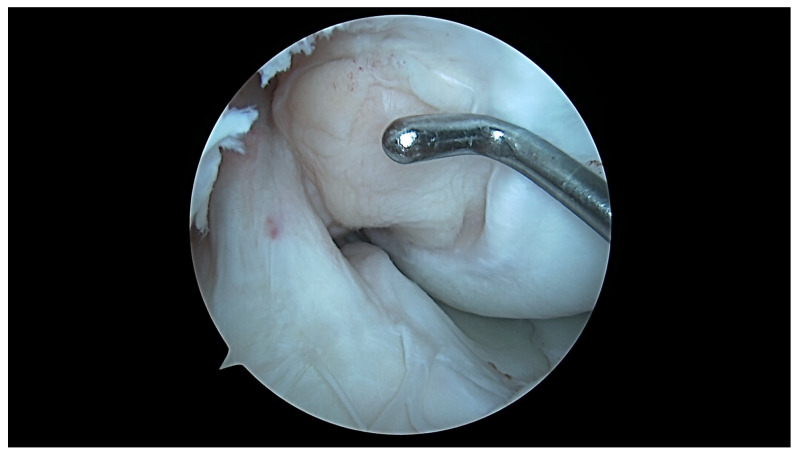
ICN width at 90 degrees: U-shaped notch type.

**Figure 10:**
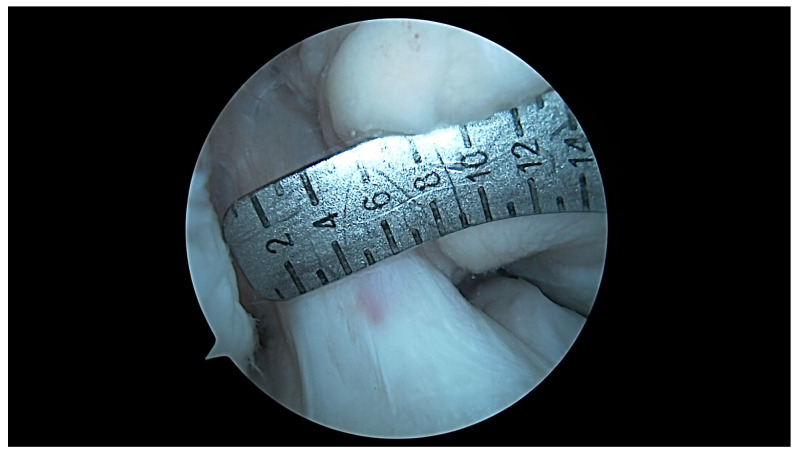
ACL width at 90 degrees flexion: U-shaped notch type.

In a knee with a U-shaped type of notch, there is a low correlation between the ACL size and the aICN area, although the ACL area is higher in this type. The internal meniscus lesions are more common in the U-shaped notch type, and this type of notch is more common in males. This might suggest different biomechanics of the knee derived from the anatomy and might suggest that U-shaped type knees are more ACL dependent, more constrained by a strong ACL, and more prone to internal meniscal lesions (Figure 7, Figure 8, Figure 9 and Figure 10).

### 4.2. A/W Shaped Notch

**Figure 11:**
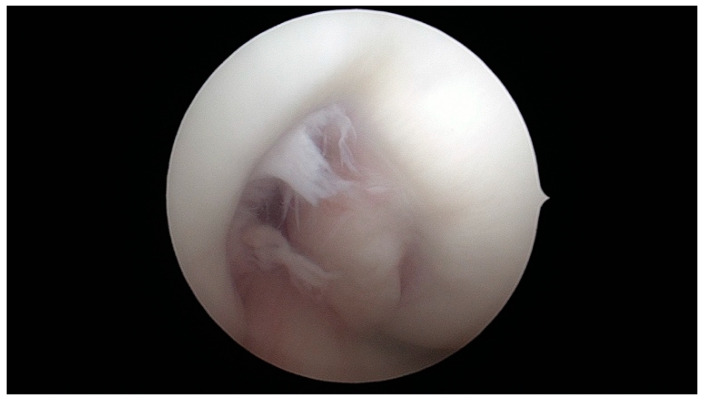
A-type notch.

**Figure 12:**
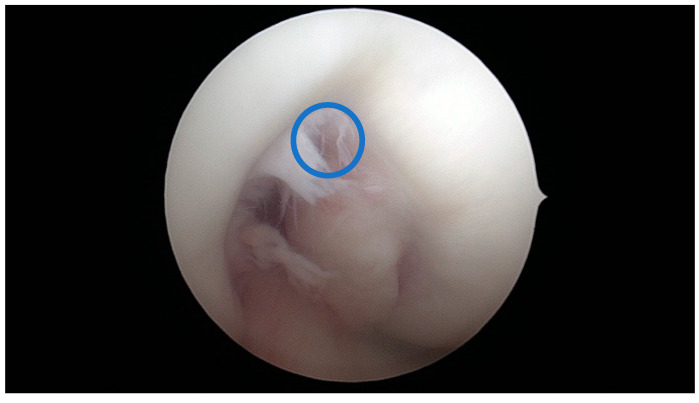
A-type notch; blue circle = aICN area measured on the MRI.

In a notch with a W/A shape, the ACL area is strongly/very strongly correlated with the anterior ICN area. External meniscus lesions were more frequent in W-shaped notches; however, we found no external meniscal lesions in A-shaped notches. This might suggest different biomechanics in this type of notch that are significantly different from the U-shaped notch-type knees. Importantly, we found that a narrow anterior notch (A/W) was correlated with a smaller native ACL size, and this might lead to graft impingement and the need for notch-plasty during ACL reconstruction due to a bigger graft than the aICN area (Figure 11 and Figure 12).

Geometric morphometry has been widely studied in patients with ACL injuries [2]. Herein, we included cases where no ACL lesions were observed using MRI, as well as cases with no arthritic changes and no previous surgery around the knee.

A recent study of knee arthroscopic surgeries described the distinct anatomical structure and connective tissue (typical glistening white surface with transversely oriented fibers of fibro-collagenous tissue with intervening blood vessels) lining the intercondylar notch’s roof, suggesting a strong association between the intercondylar fossa and the functioning of the anterior cruciate ligament [29]. This study concluded that it may protect the reconstructed ACL from graft abrasion. Thus, future research should investigate this finding in relation to the notch anatomy that we have studied here.

### 4.3. Limitations of the Study

The results of this study should be considered in the context of its limitations. It is a cross-sectional study with retrospective inclusion and lacks the inter-observer agreement. The number of studied patients was not large; this is a statistical limitation. This study does not correlate the morphological measurements with the knee phenotypes or coronal plane alignment of the knee. The technical limitation is the precision of the measurements, which derives from the resolution of the 1.5 Tesla imaging and the precision of the overlap of the axial section with the notch. Another limitation was the exclusion of the weights of the studied patients, although the BMIs of included participants were within normal limits (BMI < 30), representative of the general population.

The study is retrospective. We did not follow the patients or document further injuries. This is indeed a very interesting aspect that can be studied, and we will consider doing so in future studies. This might give very useful clinical information and impact clinical practice and prevention protocols.

## 5. Conclusions

In summary, our study provides evidence that the anterior cruciate ligament sectional area is strongly correlated with the anterior notch area when the notch shape is A or W. A thick anterior cruciate ligament is correlated with more internal meniscus lesions. The A-type notch has fewer external meniscus lesions than the W and U types. In the future, studying the notch anatomy in relation to other morphological aspects of the knee might result in a better understanding of lesion patterns and surgical decisions.

## Figures and Tables

**Table 1:** Descriptive statistics.

Parameter	Total N = 65	Male Gender N = 33	Female Gender N = 32	*p*-Value
Age, years	35 (18–50)	34 (18–50)	35 (18–50)	0.680
ACL area, cm^2^	0.48 (0.2–0.8)	0.55 (0.3–0.8)	0.41 (0.2–0.7)	0.0002
aICN area, cm^2^	0.75 (0.15–3.60)	0.96 (0.22–3.60)	0.52 (0.15–1.68)	0.0008
ICN width, mm	22.23 (12.80–31.00)	23.87 (19.00–31.00)	20.53 (12.80–28.00)	<0.0001
ICN A form, N (%)	23 (35.38%)	6 (26.09%)	17 (73.91%)	0.004
ICN W form, N (%)	17 (26.15%)	8 (47.05%)	9 (52.95%)	0.720
ICN U form, N (%)	25 (38.46%)	19 (76%)	6 (24%)	0.020
LTI, °	19.15 (3.00–31.00)	21.58 (10.00–30.00)	16.66 (3.00–31.00)	0.0009

Abbreviations: ACL, anterior cruciate ligament; ICN, intercondylar notch; LTI, lateral trochlear inclination; aICN, anterior intercondylar notch. A *p*-value less than 0.05 was considered statistically significant.

**Table 2:** Bivariate analysis of associations between intercondylar notch area and anterior cruciate ligament area.

Intercondylar Notch	ACL Area	aICN Area	Rho	*p*-Value
Total group (N = 65)				
A-shape (n_1_ = 23; 35.4%), cm^2^	0.40 (0.20–0.80)	0.47 (0.15–0.95)	0.820	<0.001
W-shape (n_2_ = 17; 26.2%), cm^2^	0.40 (0.30–0.80)	0.37 (0.15–0.81)	0.608	0.010
U-shape (n_3_ = 25; 38.5%), cm^2^	0.50 (0.20–0.80)	1.16 (0.57–3.60)	0.416	0.036

Abbreviations: ACL, anterior cruciate ligament; aICN, anterior intercondylar notch; rho, Sperman’s rho coefficient. A *p*-value less than 0.05 was considered statistically significant.

**Table 3:** Comparative analysis of the parameters measured with regard to intercondylar notch shape.

Parameter	A-Shape (N = 23)	W-Shape (N = 17)	U-Shape (N = 25)	*p*-Value	Post-Hoc
Age, median, years	33	32	37	0.877	-
ACL area, cm^2^	0.40 (0.20–0.80)	0.40 (0.30–0.80)	0.50 (0.20–0.80)	0.011	A-U
aICN area, cm^2^	0.47 (0.15–0.95)	0.37 (0.15–0.81)	1.16 (0.57–3.60)	<0.001	A-U, W-U
ICN width, mm	22 (13–28)	22 (15–26)	24 (17–31)	0.001	A-U, W-U
LTI, °	18 (7–30)	22 (7–31)	20 (3–29)	0.308	-

Abbreviations: ACL, anterior cruciate ligament; ICN, intercondylar notch; aICN, anterior intercondylar notch; LTI, lateral trochlear inclination. A *p*-value less than 0.05 was considered statistically significant.

**Table 4:** Comparative analysis of knee lesions based on the intercondylar notch shape.

Parameter	A-Shape (N = 23)	W-Shape (N = 17)	U-Shape (N = 25)	*p*-Value
Male gender, (%)	(26.1)	(47.1)	(76.0)	0.002
Internal chondral lesions, (%)	(21.7)	(5.9)	(20.0)	0.364
External chondral lesions,(%)	(0.0)	(17.6)	(12.0)	0.135
Internal meniscal lesions, (%)	(26.1)	(29.4)	(64.0)	0.014
External meniscal lesions, (%)	(0.0)	(35.3)	(16.0)	0.009
Patellar chondral lesions, (%)	(13.0)	(11.8)	(28.0)	0.291
Patella alta, (%)	(13.0)	(5.9)	(8.0)	0.715
Trochlear dysplasia, (%)	(13.0)	(17.6)	(12.0)	0.865

A *p*-value less than 0.05 was considered statistically significant.

**Table 5:** Comparative analysis of the anterior cruciate ligament area with regard to knee lesions identified via magnetic resonance imaging.

	No Lesions	Lesions Detected	*p*-Value
Internal chondral lesions	(N = 54)	(N = 11)	
	0.39 (0.23–0.69)	0.44 (0.31–0.59)	0.128
External chondral lesions	(N = 59)	(N = 6)	
	0.40 (0.23–0.69)	0.42 (0.30–0.61)	0.691
Internal meniscal injuries	(N = 38)	(N = 27)	
	0.38 (0.23–0.69)	0.46 (0.29–0.61)	0.002
External meniscal injuries	(N = 55)	(N = 10)	
	0.40 (0.23–0.69)	0.37 (0.30–0.61)	0.730
Patellar chondral lesions	(N = 53)	(N = 12)	
	0.39 (0.23–0.69)	0.47 (0.29–0.61)	0.097
Patella alta	(N = 59)	(N = 6)	
	0.40 (0.24–0.69)	0.40 (0.23–0.50)	0.650
Trochlear dysplasia	(N = 56)	(N = 9)	
	0.40 (0.23–0.69)	0.41 (0.24–0.61)	0.887

*p*-value based on the Mann–Whitney test. A *p*-value less than 0.05 was considered statistically significant. N = number of patients.

## Data Availability

No new data were created or analyzed in this study. Data sharing is not applicable to this article.

## References

[1] Whitney D.C., Sturnick D.R., Vacek P.M., DeSarno M.J., Gardner-Morse M., Tourville T.W., Smith H.C., Slauterbeck J.R., Johnson R.J., Shultz S.J. (2014). Relationship between the risk of suffering a first-time noncontact ACL injury and geometry of the femoral notch and ACL: A prospective cohort study with a nested case-control analysis. Am. J. Sports Med..

[2] Wang P., Gao F., Sun W., Li Z., Wu X., Shi L., Xu X., Li T., Fan X., Li C. (2022). Morphometric characteristics of the knee are associated with the injury of the meniscus. J. Orthop. Surg. Res..

[3] Jaibaji R., Khaleel F., Jaibaji M., Volpin A. (2023). Outcomes of Meniscal Repair in Patients Aged 40 and Above: A Systematic Review. J. Clin. Med..

[4] Iijima H., Shimoura K., Aoyama T., Takahashi M. (2018). Biomechanical characteristics of stair ambulation in patients with knee OA: A systematic review with meta-analysis toward a better definition of clinical hallmarks. Gait Posture.

[5] Migliorini F., Schäfer L., Bell A., Weber C.D., Vecchio G., Maffulli N. (2023). Meniscectomy is associated with a higher rate of osteoarthritis compared to meniscal repair following acute tears: A meta-analysis. Knee Surg. Sports Traumatol. Arthrosc..

[6] Laible C., Stein D.A., Kiridly D.N. (2013). Meniscal repair. J. Am. Acad. Orthop. Surg..

[7] Wang C.-K., Lin L.-C., Sun Y.-N., Lai C.-S., Chen C.-H., Kao C.-Y. (2021). Computer-Assisted System in Stress Radiography for Anterior Cruciate Ligament Injury with Correspondent Evaluation of Relevant Diagnostic Factors. Diagnostics.

[8] Gornitzky A.L., Lott A., Yellin J.L., Fabricant P.D., Lawrence J.T., Ganley T.J. (2016). Sport-Specific Yearly Risk and Incidence of Anterior Cruciate Ligament Tears in High School Athletes: A Systematic Review and Meta-analysis. Am. J. Sports Med..

[9] Heard W.M., VanSice W.C., Savoie F.H. (2015). Anterior cruciate ligament tears for the primary care sports physician: What to know on the field and in the office. Phys. Sportsmed..

[10] Smith H.C., Vacek P., Johnson R.J., Slauterbeck J.R., Hashemi J., Shultz S., Beynnon B.D. (2012). Risk factors for anterior cruciate ligament injury: A review of the literature. Part 1: Neuromuscular and anatomic risk. Sports Health.

[11] Javed Awan M., Mohd Rahim M.S., Salim N., Mohammed M.A., Garcia-Zapirain B., Abdulkareem K.H. (2021). Efficient Detection of Knee Anterior Cruciate Ligament from Magnetic Resonance Imaging Using Deep Learning Approach. Diagnostics.

[12] Fontanella C.G., Belluzzi E., Pozzuoli A., Scioni M., Olivotto E., Reale D., Ruggieri P., De Caro R., Ramonda R., Carniel E.L. (2022). Exploring Anatomo-Morphometric Characteristics of Infrapatellar, Suprapatellar Fat Pad, and Knee Ligaments in Osteoarthritis Compared to Post-Traumatic Lesions. Biomedicines.

[13] Weninger P., Thallinger C., Chytilek M., Hanel Y., Steffel C., Karimi R., Feichtinger X. (2023). Extracorporeal Shockwave Therapy Improves Outcome after Primary Anterior Cruciate Ligament Reconstruction with Hamstring Tendons. J. Clin. Med..

[14] Biz C., Cigolotti A., Zonta F., Belluzzi E., Ruggieri P. (2019). ACL reconstruction using a bone patellar tendon bone (BPTB) allograft or a hamstring tendon autograft (GST): A single-center comparative study. Acta Biomed..

[15] MacDessi S.J., Griffiths-Jones W., Harris I.A., Bellemans J., Chen D.B. (2021). Coronal Plane Alignment of the Knee (CPAK) classification. Bone Jt. J..

[16] MacDessi S.J., Griffiths-Jones W., Harris I.A., Bellemans J., Chen D.B. (2020). The arithmetic HKA (aHKA) predicts the constitutional alignment of the arthritic knee compared to the normal contralateral knee. Bone Jt. Open..

[17] Calliess T., Bauer K., Stukenborg-Colsman C., Windhagen H., Budde S., Ettinger M. (2017). Psi kinematic versus non-PSI mechanical alignment in total knee arthroplasty: A prospective, randomized study. Knee Surg. Sports Traumatol. Arthrosc..

[18] Hutt J.R.B., LeBlanc M.-A., Massé V., Lavigne M., Vendittoli P.-A. (2016). Kinematic TKA using navigation: Surgical technique and initial results. Orthop. Traumatol. Surg. Res..

[19] Bali K., Walker P., Bruce W. (2012). Custom-fit total knee arthroplasty: Our initial experience in 32 knees. J. Arthroplast..

[20] Howell S.M., Papadopoulos S., Kuznik K.T., Hull M.L. (2013). Accurate alignment and high function after kinematically aligned TKA performed with generic instruments. Knee Surg. Sports Traumatol. Arthrosc..

[21] Almaawi A.M., Hutt J.R.B., Masse V., Lavigne M., Vendittoli P.-A. (2017). The impact of mechanical and restricted kinematic alignment on knee anatomy in total knee arthroplasty. J. Arthroplast..

[22] McEwen P., Balendra G., Doma K. (2019). Medial and lateral gap laxity differential in computer-assisted kinematic total knee arthroplasty. Bone Jt. J..

[23] Waterson H.B., Clement N.D., Eyres K.S., Mandalia V.I., Toms A.D. (2016). The early outcome of kinematic versus mechanical alignment in total knee arthroplasty: A prospective randomised control trial. Bone Jt. J..

[24] Griffiths-Jones W., Chen D.B., Harris I.A., Bellemans J., MacDessi S.J. (2021). Arithmetic hip-knee-ankle angle (aHKA): An algorithm for estimating constitutional lower limb alignment in the arthritic patient population. Bone Jt. Open.

[25] Victor J.M.K., Bassens D., Bellemans J., Gürsu S., Dhollander A.A.M., Verdonk P.C.M. (2014). Constitutional varus does not affect joint line orientation in the coronal plane. Clin. Orthop. Relat. Res..

[26] Hirschmann M.T., Moser L.B., Amsler F., Behrend H., Leclerq V., Hess S. (2019). Functional knee phenotypes: A novel classification for phenotyping the coronal lower limb alignment based on the native alignment in young non-osteoarthritic patients. Knee Surg. Sports Traumatol. Arthrosc..

[27] Hutt J., Massé V., Lavigne M., Vendittoli P.-A. (2016). Functional joint line obliquity after kinematic total knee arthroplasty. Int. Orthop..

[28] Chow J.C., Breslauer L. (2017). The use of intraoperative sensors significantly increases the patient-reported rate of improvement in primary total knee arthroplasty. Orthopedics.

[29] Nag H.L., Jain G., Vijayakumar V., Jacob T.G., Sonai M., Lalwani S. (2021). Femoral Intercondylar Notch: Gross Anatomy and Histology of the Connective Tissue Lining of its Roof: A Pilot Study. Surg. Radiol. Anat..

